# Physical and dosimetric characterization of thermoset shape memory bolus developed for radiotherapy

**DOI:** 10.1002/mp.14516

**Published:** 2020-10-22

**Authors:** Takahiro Aoyama, Koichiro Uto, Hidetoshi Shimizu, Mitsuhiro Ebara, Tomoki Kitagawa, Hiroyuki Tachibana, Kojiro Suzuki, Takeshi Kodaira

**Affiliations:** ^1^ Department of Radiation Oncology Aichi Cancer Center 1‐1 Kanokoden, Chikusa‐Ku Nagoya Aichi 464‐8681 Japan; ^2^ Graduate School of Medicine Aichi Medical University 1‐1 Yazako‐karimata Nagakute Aichi 480‐1195 Japan; ^3^ Research Center for Functional Materials National Institute for Materials Science 1‐1 Namiki Tsukuba Ibaraki 305‐0044 Japan; ^4^ Department of Radiology Aichi Medical University 1‐1 Yazako‐karimata Nagakute Aichi 480‐1195 Japan

**Keywords:** bolus, poly‐ε‐caprolactone, radiotherapy, shape memory, thermoset

## Abstract

**Purpose:**

We developed a thermoset shape memory bolus (shape memory bolus) made from poly‐ε‐caprolactone (PCL) polymer. This study aimed to investigate whether the shape memory bolus can be applied to radiotherapy as a bolus that conformally adheres to the body surface, can be created in a short time, and can be reused.

**Methods:**

The shape memory bolus was developed by cross‐linking tetrabranch PCL with reactive acrylate end groups. Dice similarity coefficient (DSC) was used to evaluate shape memory characterization before deformation and after restoration. In addition, the degree of adhesion to the body surface and crystallization time were calculated. Moreover, dosimetric characterization was evaluated using the water equivalent phantom and an Alderson RANDO phantom.

**Results:**

The DSC value between before deformation and after restoration was close to 1. The degree of adhesion of the shape memory bolus (1.9%) was improved compared with the conventional bolus (45.6%) and was equivalent to three‐dimensional (3D) printer boluses (1.3%–3.5%). The crystallization time was approximately 1.5 min, which was clinically acceptable. The dose calculation accuracy, dose distribution, and dose index were the equivalent compared with 3D boluses.

**Conclusion:**

The shape memory bolus has excellent adhesion to the body surface, can be created in a short time, and can be reused. In addition, the shape memory bolus needs can be made from low‐cost materials and no quality control systems are required for individual clinical departments, and it is useful as a bolus for radiotherapy.

## 1 INTRODUCTION

Sufficient dose to the skin surface is difficult to prescribe in high‐energy x‐ray and electron beam used in radiotherapy because of the build‐up effect.^1^ Therefore, if the tumor is on the skin surface, a bolus is usually used to increase the surface dose.^1^, ^2^ Conventional flat‐type bolus (conventional bolus) is useful for areas with flat body surface, such as the abdomen and back. However, in body areas with a non‐flat surface, such as the face and breast, dose distribution on the surface may not lead to significant improvements because of poor adhesion.^1^ In recent years, a patient‐specific bolus [three‐dimensional (3D) bolus] has been made using 3D printer technology to improve the degree of adhesion on non‐flat areas.^3^, ^4^, ^5^, ^6^ Improvement of dose distribution and dose index using 3D boluses compared with conventional boluses has been reported.^7^, ^8^, ^9^, ^10^, ^11^ Three‐dimensional bolus is made for several hours to several days using body surface data obtained from computed tomography (CT) images or surface scanner images of patients. There are two methods of applying the 3D bolus to radiotherapy planning. In the first method, we use the 3D bolus created in advance for CT simulation (CTs).^8^, ^9^, ^11^ This method requires the 3D bolus before CTs; hence, additional examination is needed to obtain body surface data. In addition, making 3D bolus sometimes requires several days; therefore, the local control rate of the tumor may reduce owing to delayed treatment initiation.^12^ Another method is applying a virtual bolus for radiotherapy planning.^13^ This method does not affect the treatment schedule. However, the treatment accuracy will be reduced if the shape or density of the virtual bolus is different from actual 3D bolus. Furthermore, 3D boluses cannot be reused unlike conventional boluses because they are designed specifically for patients. Therefore, it is necessary to dispose them every time treatment ends, which consumes a lot of medical resources. In particular, environmental pollution during disposal can be a problem with some materials, including acrylonitrile butadiene styrene resin, when used in 3D printing.^14^ Therefore, the 3D bolus cannot be made in a short time and cannot be reused, although it has excellent adhesion to the patient's body surface.

A thermoplastic mask made of poly‐ε‐caprolactone (PCL) using the immobilization system adheres to the body surface within a few minutes.^15^, ^16^, ^17^ Thus, the thermoplastic boluses using the materials of immobilization systems are now available in the market^18^; however, the shape cannot be restored and reused because the thermoplastic bolus is not chemically cross‐linked.

Characteristics such as excellent adhesion, short time, and reuse are important in terms of clinical and environmental aspects; however, there is no bolus that has all these characteristics. We developed a thermoset shape memory bolus using PCL polymer with shape memory characteristics and investigated whether the shape memory bolus can be applied to radiotherapy that conformally adheres to the body surface, can be created in a short time, and can be reused.

## 2 MATERIALS AND METHODS

**Fig. 1:**
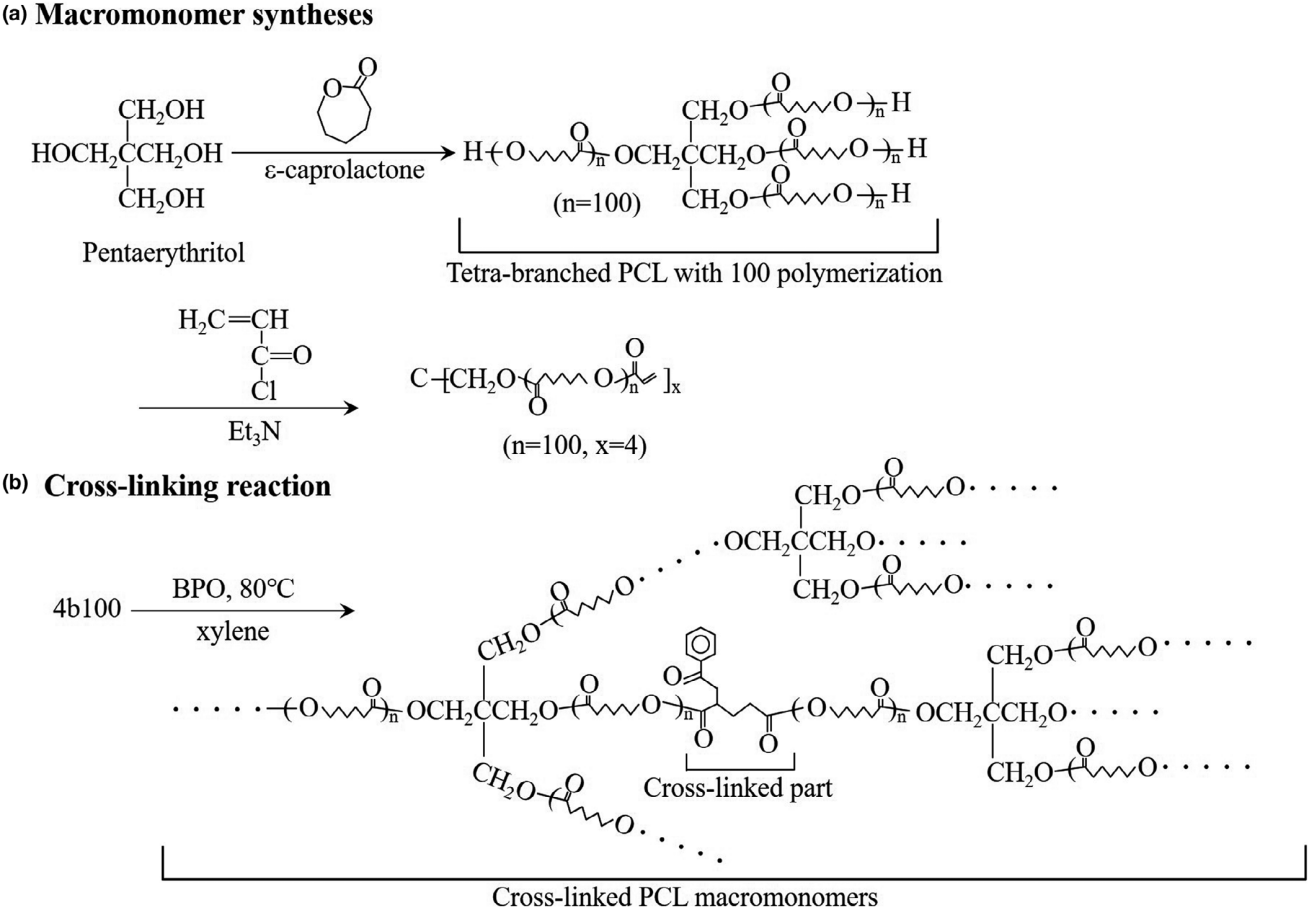
Schematic illustrations of (a) macromonomer synthesis and (b) cross‐linking reaction.

Our group has been conducting research on thermoset PCL polymers with shape memory characteristics. In this study, the shape memory bolus was developed by changing the molecular weights of the thermoset PCL used in a previous study.^19^ The procedure for developing the shape memory bolus from thermoset PCL is presented in Section 2.A and Fig. 1.

### 2.A Development of the shape memory bolus

#### 2.A.1 Synthesis of branched PCL macromonomer

The shape memory bolus was developed by cross‐linking tetrabranch PCL with acrylate end groups (macromonomer) according to a previously reported protocol.^20^, ^21^ Briefly, tetrabranch PCL was synthesized by ring‐opening polymerization of ε‐caprolactone [Tokyo Chemical Industry (TCI) Co., Ltd, Tokyo, Japan] with pentaerythritol (TCI Co., Ltd, Tokyo, Japan) as initiators. Then, acryloyl chloride (TCI Co., Ltd, Tokyo, Japan) was reacted with the hydroxyl end group of the branched chains. The structures and molecular weights were estimated using ^1^H nuclear magnetic resonance spectroscopy (JEOL, Tokyo, Japan). The average degree of polymerization of tetrabranch PCL was 100 [Fig. 1(a)]. The tetrabranch PCL with average degrees of polymerization of 100 was abbreviated as 4b100.

#### 2.A.2 Fabrication of cross‐linked PCL macromonomers

**Fig. 2:**
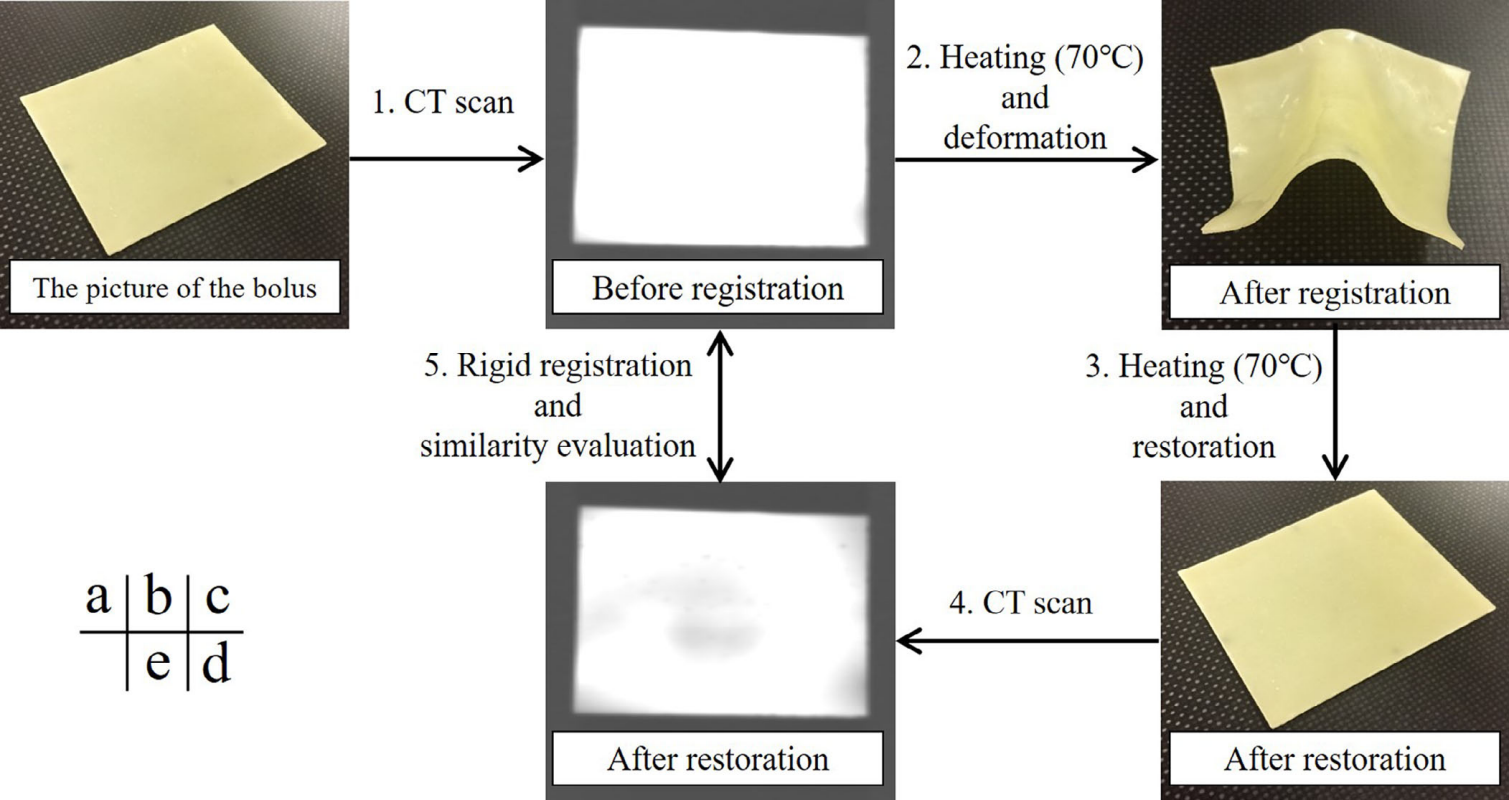
Evaluation of shape memory characteristics of the shape memory bolus before deformation and after restoration. [Color figure can be viewed at wileyonlinelibrary.com]

The tetrabranch PCL macromonomers were dissolved at 50 wt% in xylene containing 1.5 wt% (against polymer) benzoyl peroxide (BPO; Sigma‐Aldrich, St. Louis, MO, USA). The solution was injected between glass slides with a 0.2‐cm thick Teflon spacer. Then, thermal polymerization was performed at 80°C overnight to obtain the cross‐linked PCL [Fig. 1(b)]. From these processes, the shape memory bolus with a thickness of 0.15 cm was developed [Fig. 2(a)].

### 2.B Physical characterization of the shape memory bolus

#### 2.B.1 Evaluation of shape memory characteristics

The shape of the developed bolus before deformation and after restoration was evaluated to investigate the shape memory characteristics. The Aquilion LB CT system (Canon Medical Systems, Tochigi, Japan) was used under the following conditions: tube voltage = 120 kV, tube current = 300 mA, matrix size = 512 × 512 pixels, field of view = 550 mm, and slice thickness = 1 mm. The evaluation procedure is as follows: (a) contouring from CT images of the shape memory bolus using RayStation version 6.2 (RaySearch Laboratories, Stockholm, Sweden) [Fig. 2(b)], (b) deforming the shape memory bolus according to the nose of an Alderson RANDO (RANDO) phantom (The Phantom Laboratory, Salem, NY, USA) after softening by immersing it in 70°C water for 30 s and cooling at room temperature [Fig. 2(c)], (c) shape restoration by immersing in 70°C water for 30 s [Fig. 2(d)], (d) CT scan and contouring of the restored shape memory bolus (Fig. 2(e)], and (e) similarity evaluation of 2 contours after rigid registration using RayStation. To evaluate the reproducibility of the shape memory bolus, the similarity before deformation and after restoration was assessed using the dice similarity coefficient (DSC) and overlapping index (OI), which were calculated as follows:

(1) $$DSC\;=\;\frac{2\left|A\cap B\right|}{\left|A\right|+\left|B\right|}$$

(2) $$OI\;=\;\frac{\left|A\cap B\right|}{\left|A\right|}$$

where A and B are the two structures that were evaluated. DSC and OI can range from 0 (no overlap) to 1 (complete overlap). A is the structure of the bolus before deformation, and B is the structure after restoration. Procedures from 1 to 5 were performed three times using different samples, and the average values ± standard deviation was calculated. In addition, the DSC value was calculated by equation 1 when only the position was changed without deformation.

#### 2.B.2 Evaluation of the degree of adhesion and crystallization time of the shape memory bolus

**Fig. 3:**
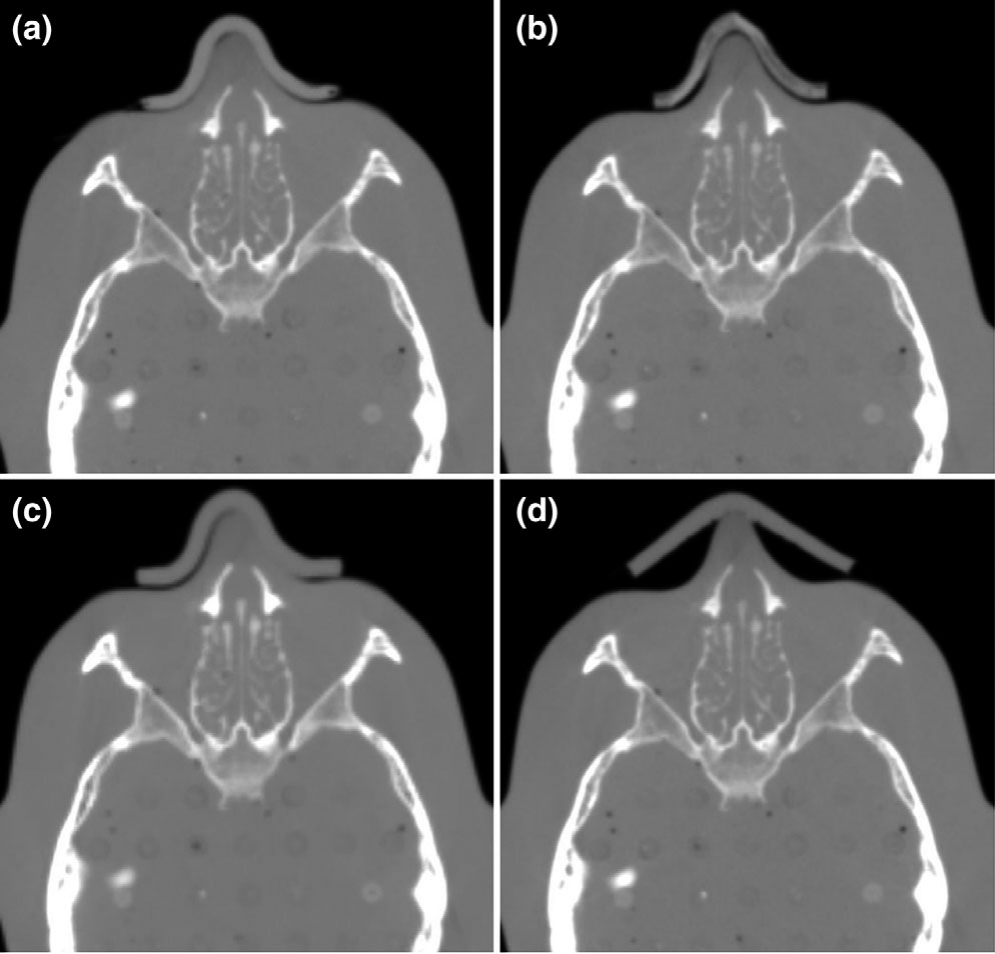
Computed tomography images of the RANDO phantom with the shape memory bolus (a), three‐dimensional (3D) bolus_PLA_ (b), 3D bolus_PU_ (c), and conventional bolus (d).

**Fig. 4:**
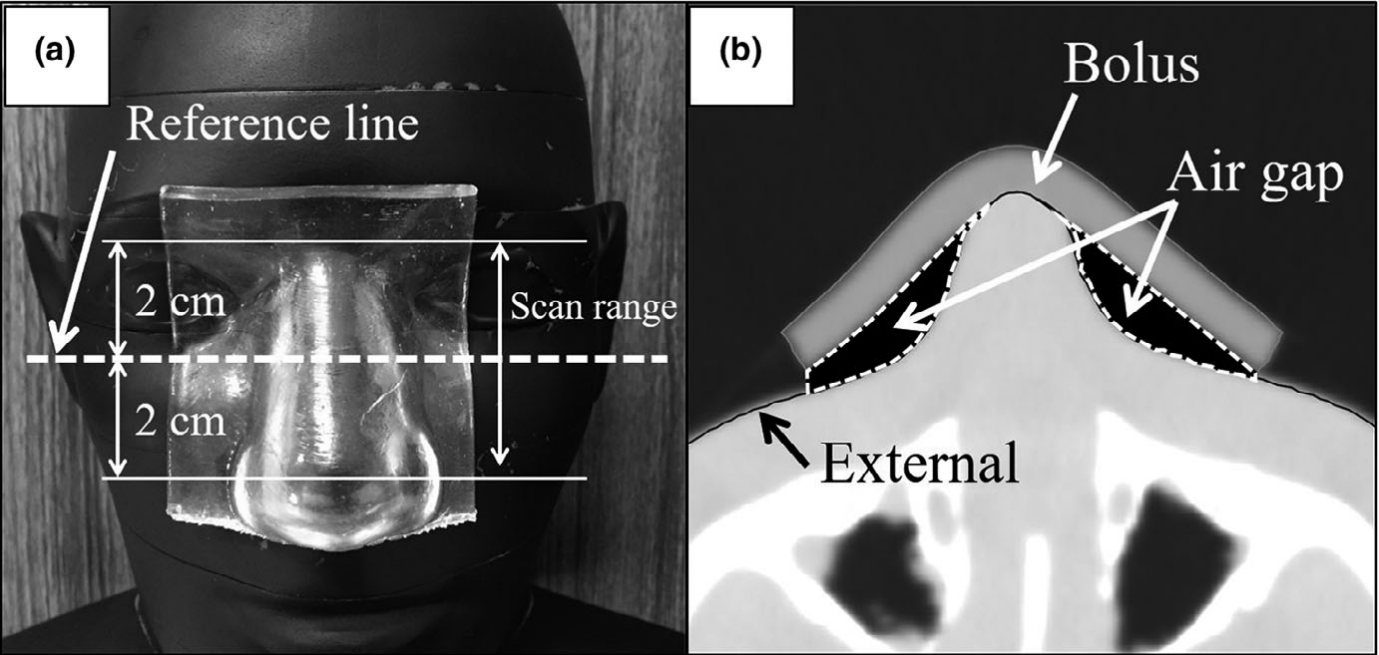
(a) Scanning range of the computed tomography image and (b) contour of the bolus and air gap.

The RANDO phantom with the shape memory bolus was scanned [Fig. 3(a)] to evaluate the degree of adhesion of the shape memory bolus to the body surface. Using CT images with scanning range of ±2 cm from the reference line, the air gap between the RANDO phantom and the bolus was manually contoured (Fig. 4). The degree of adhesion was calculated by the following formula．

(3) $$Degree\;of\;adhesion\left[\text{\%}\;\right]\;=\;\frac{Volume\;of\;the\;air\;gap}{Volume\;of\;the\;bolus}\;\times \;100$$

In addition, a 3D bolus [Fig.3(b)] was generated using the NJB‐300W personal 3D printer (Ninjabot, Shizuoka, Japan) with a polylactic acid (PLA) filament (3D bolus_PLA_). The marching cube algorithm was applied while converting from DICOM to STL, and the Laplacian smoothing filter was utilized to smoothen the polygonal mesh with the RAD modeler (tetraface Inc., Tokyo, Japan), which is a 3D‐modeling software. The printing parameters are presented in Table I. Moreover, another commercial 3D bolus (Adjust polymer, Shizuoka, Japan) [Fig. 3(c)] made from polyurethane resin (PU) was created (3D bolus_PU_). The degree of adhesion was also calculated for 3D bolus_PLA_, 3D bolus_PU_, and conventional bolus (MTCB405S: CIVCO Medical Solutions, Iowa, USA) [Fig. 3(d)].

**Table I:** Printing parameters of three‐dimensional bolus_PLA_.

Settings	Values
Bed temperature	0°C
Extruder temperature	200°C
Layer height	0.3 mm
First layer height	0.3 mm
Vertical shells	5
Horizontal shells	5
Speed of print movement (perimeter)	30 mm/s
Speed of print movement (external perimeter)	15 mm/s
Speed of print movement (infill)	30 mm/s
First layer print movements speed	15 mm/s
Fill density	100%
Fill pattern	Rectilinear
Fill angle	45°
Nozzle diameter	0.4 mm
Filament diameter	1.75 mm

Furthermore, the crystallization time after softening and stopping the heating was also measured.

### 2.C Dosimetric characterization of the shape memory bolus

The water equivalent phantom was used to evaluate the dose calculation accuracy of the shape memory bolus in the treatment planning system. Next, treatment plans were compared using the RANDO phantom with virtual nasal cavity cancer to evaluate the dose distributions between the shape memory bolus and the 3D boluses.

#### 2.C.1 Dosimetry using the water equivalent phantom

**Fig. 5:**
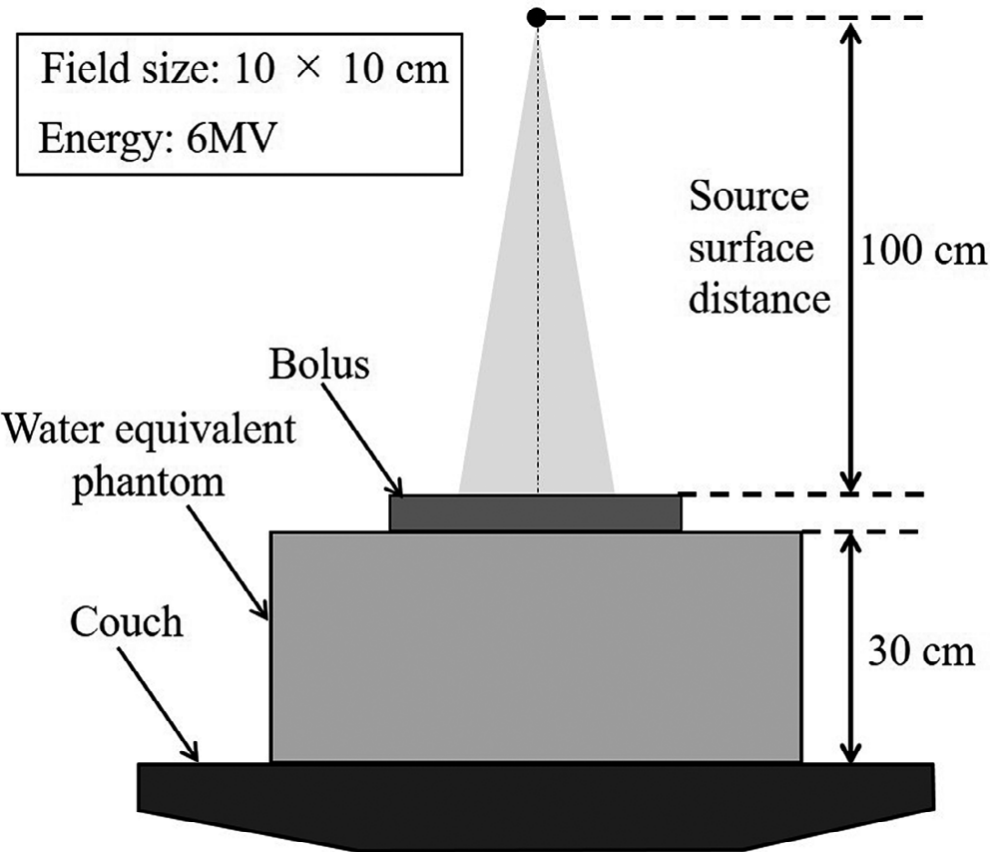
Geometric arrangement of the dose measurement after passing through the bolus.

Four treatment plans using water equivalent phantoms and four types of boluses (shape memory bolus, 3D bolus_PLA,_ 3D bolus_PU_, and conventional bolus) were created to evaluate the dose calculation accuracy after passing through the bolus. The shape memory bolus had a physical density of 1.15 g/cm^3^ and thickness of 0.45 cm with three layers. The physical densities of the 3D bolus_PLA_, 3D bolus_PU_, and conventional bolus were 1.25, 1.03, and 1.03 g/cm^3^, respectively, with a thickness of 0.5 cm in all the boluses. These densities were examined using the objects tested. The bolus was placed on a 30 cm stack of solid water HE (nominal density = 1.032 ± 0.005 g/cm^3^) (Sun Nuclear Co., Melbourne, Australia) on a couch top, and the source surface distance was set at 100 cm (Fig. 5). The x‐ray energy was 6 MV using TrueBeam (Varian Medical Systems, California, USA), the field size was 10 cm × 10 cm, and MU was 100. The percentage depth dose (PDD) on the central axis was obtained using a plane‐parallel chamber (PTW34001) (PTW, Freiburg, German). In addition, absolute doses of 1, 5, 10, and 20 cm in depth were measured using a farmer chamber (PTW30013) (PTW, Freiburg, Germany). Dose measurements were performed five times, and the average of the five measurements was taken as the measurement value. Dose calculation was performed with the same geometric arrangement as dose measurement with RayStation. Four types of boluses were contoured and overwritten with the physical density of each bolus.^22^ Collapsed cone convolution version 3.4 was used as the dose calculation algorithm with RayStation, and the dose calculation grid size was 0.1 cm. The PDD on the central axis obtained using the treatment planning system was compared with the measured value of the plane‐parallel chamber. The PDD curves were normalized at a depth of 10 cm, and the wall thickness of the plane‐parallel chamber was considered by shifting the measured PDD. In addition, the dose difference between the calculated doses and the measured doses with the plane‐parallel and farmer chamber was evaluated as follows:

(4) $$Dose\;difference\;\left[\text{\%}\;\right]\;=\;\frac{Measured\;dose\;‐\;Calculated\;dose}{Calculated\;dose}\;\times 100$$

The depth‐scaling factor was applied by overwriting with 1.03, which is the density of the water equivalent phantom, and the applied fluence‐scaling factor was 1.

#### 2.C.2 Comparison of dose distribution and dose index using the RANDO phantom

**Fig. 6:**
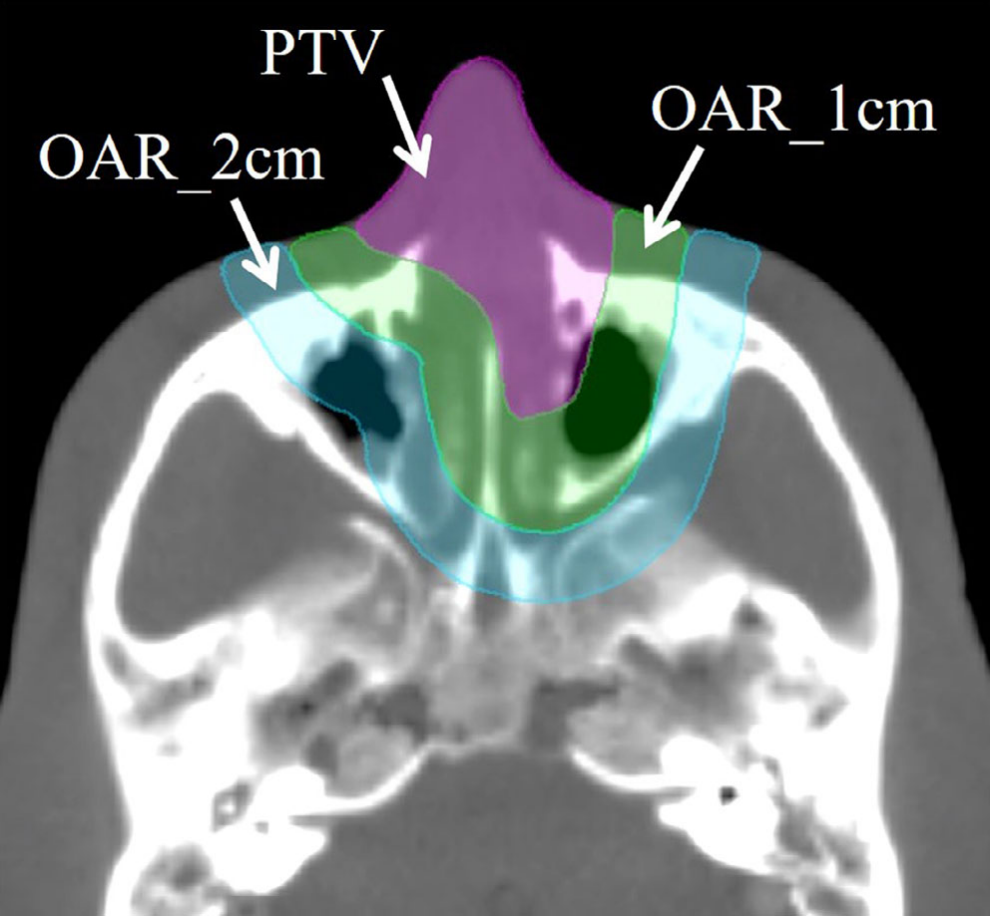
Illustration of the virtual planning target volume and organ at risk (OAR_1cm and OAR_2cm). [Color figure can be viewed at wileyonlinelibrary.com]

To investigate whether the shape memory bolus can produce the equivalent dose distribution and dose index as the 3D boluses, three treatment plans were created for virtual nasal cavity cancer. Using CT images of the shape memory bolus, the 3D bolus_PLA_, and the 3D bolus_PU_, treatment plans of 200 cGy/fraction were created for a virtual planning target volume (PTV: 26.2 mL) in the nasal cavity region of the RANDO phantom. Rings with 1 or 2 cm away from the target edge were contoured as organ at risk (OAR_1cm, 59.3 mL; OAR_2cm, 104.9 mL) (Fig. 6). Two types of plans were created: single static field with field size of 4 cm × 4 cm and gantry angle of 0° and single arc volumetric‐modulated arc therapy (VMAT) with a gantry angle of 181° to 179°. The MU value was scaled to include 95% volume of the PTV at 200 cGy (D_95%_ = 200 cGy). Under these conditions, D_98%_, D_50%_, and D_2%_ of PTV and D_mean_ and D_1%_ of OARs were calculated from dose–volume histogram (DVH).

## 3 RESULTS

### 3.A Physical characterization of the shape memory bolus

#### 3.A.1 The DSC value before deformation and after restoration

The shape memory characteristics were evaluated using the DSC value before deformation and after restoration by the method presented in Section 2.B.1. The calculated DSC value was 0.979 ± 0.006. The DSC value was 0.975 ± 0.008 when only the position was changed without deformation. When the DSC values before deformation and after restoration were very close to 1 and almost equal to that without deformation, the shape memory bolus had an almost perfect shape memory characteristic. Additionally, because the OI value was 0.977 ± 0.011, there was no volume change.

#### 3.A.2 Degree of adhesion and crystallization time of the shape memory bolus

The degree of adhesion of the boluses calculated by the method presented in Section 2.B.2 is 1.9% (shape memory bolus), 3.5% (3D bolus_PLA_), 1.3% (3D bolus_PU_), and 45.6% (conventional bolus) (Table II). The adhesion of the shape memory bolus was improved compared with the conventional bolus and was equivalent to the 3D bolus_PLA_ and the 3D bolus_PU_. In addition, the crystallization time of the shape memory bolus after softening was 1.5 min at room temperature.

**Table II:** The degree of adhesion calculated by the volume of boluses and air gap; $Degree\;of\;adhesion\left[\text{\%}\;\right]\;=\;\frac{Volume\;of\;air\;gap}{Volume\;of\;the\;bolus}\;\times 100$.

Bolus type	Volume of bolus (cc)	Volume of air gap (cc)	Degree of adhesion (%)
Shape memory bolus	20.85	0.40	1.9
3D bolus_PLA_	21.42	0.75	3.5
3D bolus_PU_	21.50	0.27	1.3
Conventional bolus	22.68	10.35	45.6

### 3.B Dosimetric characterization of the shape memory bolus

#### 3.B.1 Dosimetry using the water equivalent phantom

**Fig. 7:**
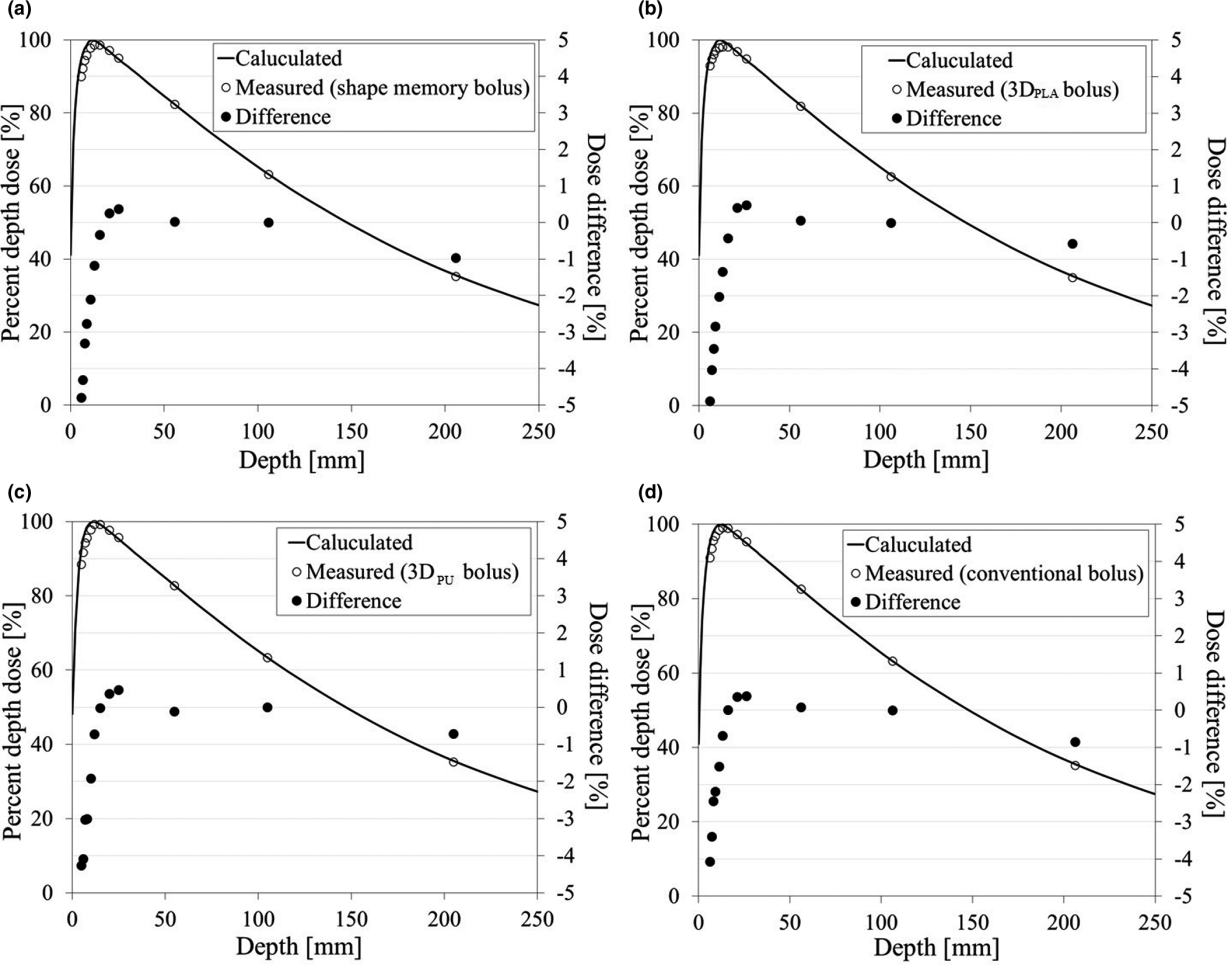
Comparison of the calculated percentage depth dose curve (line), the measured values (white circle), and dose difference (black circle) with the shape memory bolus (a), three‐dimensional (3D) bolus_PLA_ (b), 3D bolus_PU_ (c), and conventional bolus (d).

The calculated PDD curve and the measured values using the plane‐parallel ion chamber and their differences are presented in Fig. 7. For four boluses, the dose differences of calculated and measured values were within ±5.0% (range, −4.8% to 0.7%) in the build‐up region and within ±1.5% (range, −1.2% to 0.5%) after the build‐up region [Figs. 7(a)–7(d)]. The dose differences between the calculated dose and the measured dose using a farmer‐type ion chamber at depths of 1, 5, 10, and 20 cm are presented in Table III. The dose difference was within ±1.5% (range, −1.4% to 0.5%) in all boluses, which agreed well.

**Table III:** The dose difference of the calculated dose and measured dose at 1 cm (D_1cm_), 5 cm (D_5cm_), 10 cm (D_10cm_), and 20 cm (D_20cm_); $Dose\;difference\left[\text{\%}\;\right]\;=\;\frac{Measured\;dose‐calculated\;dose}{Calculated\;dose}\;\times 100$.

Bolus type	Dose difference (%)			
	D_1cm_	D_5cm_	D_10cm_	D_20cm_
Shape memory bolus	−1.42	0.45	0.46	−1.05
3D bolus_PLA_	−0.83	0.06	0.39	−0.82
3D bolus_PU_	−0.33	0.20	0.50	−0.39
Conventional bolus	−0.67	−0.48	−0.17	−1. 1 6

#### 3.B.2 Comparison of dose distribution and dose index using RANDO phantom

**Fig. 8:**
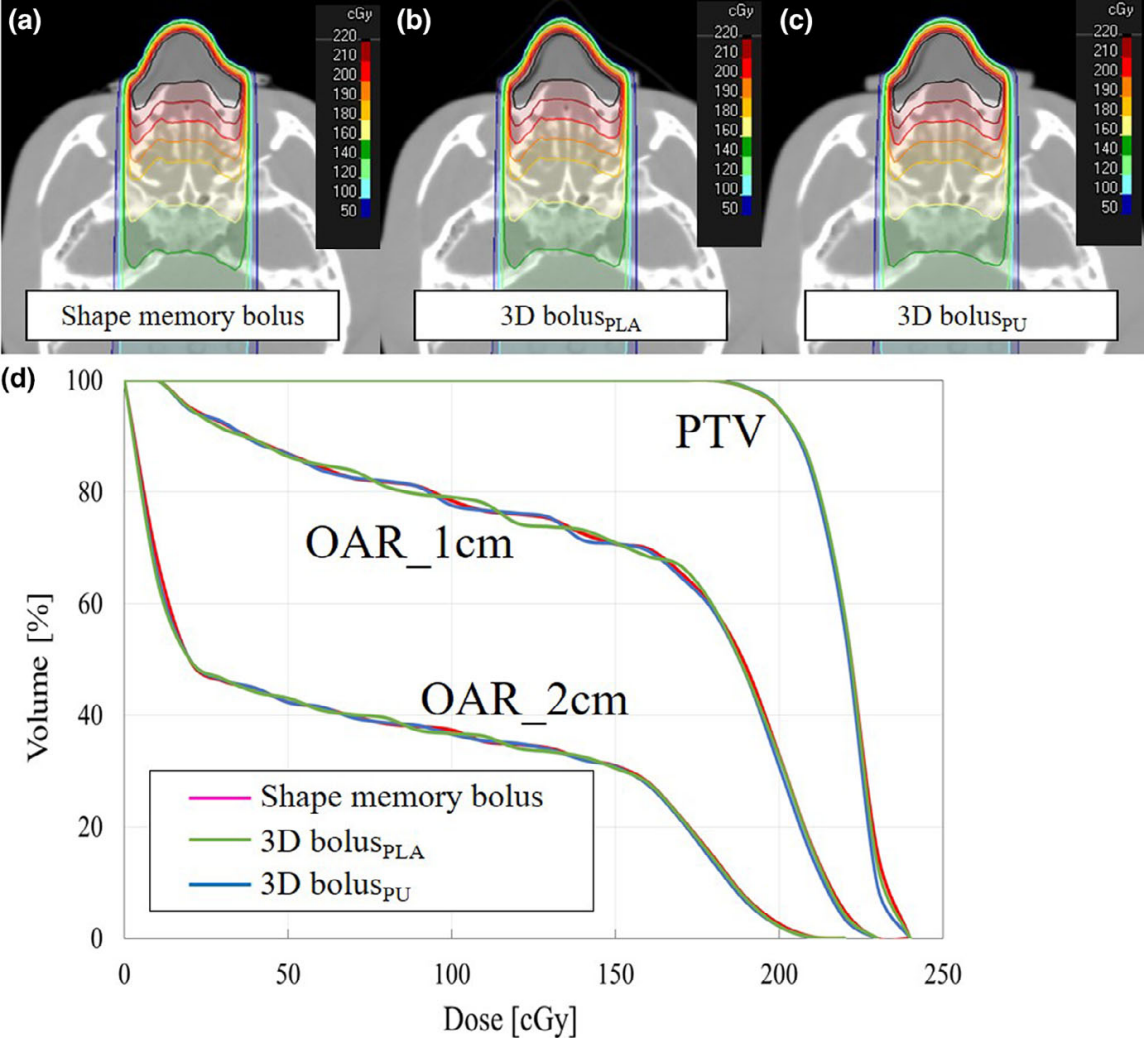
(a) Dose distribution with the shape memory bolus, (b) three‐dimensional (3D) bolus_PLA_, (c) 3D bolus_PU_, and (d) dose–volume histogram of the single static field plan for RANDO phantom. [Color figure can be viewed at wileyonlinelibrary.com]

**Fig. 9:**
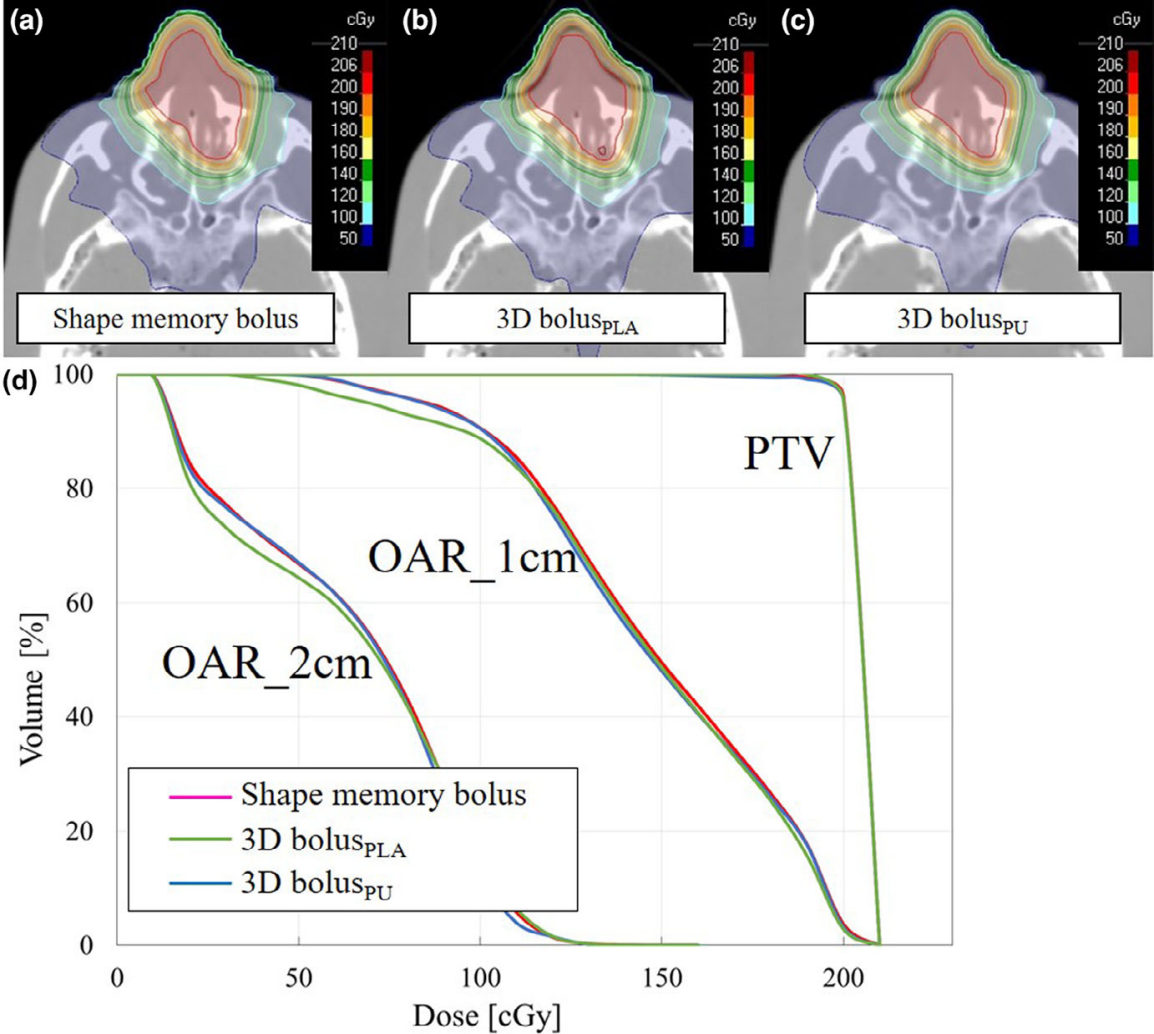
(a) Dose distribution with the shape memory bolus, (b) three‐dimensional (3D bolus_PLA_), (c) 3D bolus_PU_, and (d) dose–volume histogram of the VMAT plan for RANDO phantom. [Color figure can be viewed at wileyonlinelibrary.com]

The dose distribution and DVH of the single static field plan and VMAT plan with the shape memory bolus, 3D bolus_PLA_, and 3D bolus_PU_ are presented in Figs. 8 and 9. The dose index is presented in Table IV. The quality of the dose distribution and dose index were equivalent in all plans.

**Table IV:** Comparison of the dose index using RANDO phantom with shape memory bolus, 3D bolus_PLA_, and 3D bolus_PU_.

Irradiation technique	Bolus type	PTV (cGy)			OAR_1cm (cGy)		OAR_2cm (cGy)	
		D_98%_	D_50%_	D_2%_	D_mean_	D_1%_	D_mean_	D_1%_
Singlestatic field	Shape memory bolus	193	222	236	159	228	74	207
Singlestatic field	3D bolus_PLA_	193	221	234	158	227	74	206
Singlestatic field	3D bolus_pu_	193	222	235	158	228	74	206
VMAT	Shape memory bolus	198	203	206	149	207	66	124
VMAT	3D bolus_PLA_	197	203	205	148	207	65	124
VMAT	3D bolus_pu_	197	203	204	146	206	64	124

## 4 DISCUSSIONS

In this study, the shape memory bolus was developed using the thermoset PCL polymers and was confirmed to have almost perfect shape memory characteristics. Because the shape memory bolus has a covalent network structure after cross‐linking (i.e., chemically cross‐linked PCL), it is more chemically stable than the PCL used in thermoplastic masks. Therefore, the shape memory characteristics are not lost unless physically destroyed even if a high dose is irradiated and deformation and restoration are repeated. It can probably be used for at least 6 months to 1 yr, similar to commercially available thermoplastic masks. In addition, PCL has been approved for cleaning with 70% alcohol, as per the World Health Organization's (WHO) recommended cleaning procedures for COVID‐19.^23^, ^24^ Therefore, the shape memory bolus made of PCL can be safely reused. Even when the shape memory bolus is destroyed and discarded, there are few adverse effects on the environment owing to the biodegradability of PCL.^25^

When the average degree of polymerization of tetrabranch PCL was changed to 35 (4b35) from 100, the degree of adhesion of the 4b35 (3.4%, data not indicated) was slightly decreased compared with 4b100 (1.9%). It is considered that the deformation ability in 4b35 was reduced because the average degree of polymerization was less than that of 4b100.^21^ Meanwhile, as the average degree of polymerization is larger, it is considered that the deformation ability of the polymer was large. However, excessive polymerization might not be suitable for a bolus because chemical cross‐linking becomes insufficient and stability during heating decreases. In addition, the crystallization time also changes because of the change in the average degree of polymerization.^19^, ^20^ It is considered that the shape memory bolus made from 4b100 is optimal for the bolus because the crystallization time of 1.5 min at room temperature is not too short to mimic the shape of the body surface and be clinically acceptable.

The dose difference of the shape memory bolus in the PDD and absolute dose was within ±1.5%, excluding the build‐up region, and was the same as other boluses (Fig. 6 and Table III). It may be noted that an ionization chamber was used for dose measurement. Thus, the evaluation of surface dose might not have been performed accurately. Detailed examinations, such as the assessment of varying bolus thickness, film analysis, and Monte Carlo simulation, should be performed prior to clinical use. The treatment plan for virtual tumor using the shape memory bolus was equivalent to the 3D boluses in both dose distribution and dose index (Figs. 7 and 8 and Table IV). These results were consistent with other studies reporting that the dose calculation of 3D bolus was accurately calculated using treatment planning system and the dose distribution was improved.^7^, ^18^

The characteristics of 3D boluses depend on the creation process, the 3D printer model, type of slicer software, and setting parameters. Therefore, even if the same material is used, the same bolus cannot be created.^26^ Moreover, 3D boluses have many unclear points regarding changes during treatment period and durability and safety against radiation.^27^ Therefore, to use the 3D bolus clinically, it is necessary to build a quality control flow for each facility, which requires a lot of time and labor costs.^13^, ^27^ The shape memory bolus is advantageous as it is easy to operate because its creation procedure is similar to that of immobilization systems. In addition, PCL has long been used in thermoplastic masks for radiotherapy, and its durability and safety against radiation during the treatment period are guaranteed. Moreover, PCL polymers are inexpensive ($10/kg) compared with commercially available patient‐specific 3D boluses ($2381/piece^8^). Furthermore, PCL used in shape memory boluses has been approved by the Food and Drug Administration (FDA); hence, it is highly reliable in clinical use. Our findings confirmed that the shape memory bolus developed in this study has excellent adhesion to the body surface, can be created in a short time with low costs, can be reused, and was indicated to be useful as a bolus for radiotherapy. By contrast, once the shape memory bolus is crystallized, the shape cannot be deformed or restored unless it is reheated. Therefore, to facilitate adhesion to the patient’s body surface in the event of surface changes, reshaping after reheating is required. In addition, the shape memory bolus must be shaped by pressing it on the patient’s skin, which can be painful if there are wounds.

There are two major limitations in this study. First, because the thickness of the shape memory bolus was 0.15 cm, there was a need to stack for compensation of build‐up distance. One of the methods to increase the thickness of the bolus was the use of a thicker Teflon spacer (e.g., 3.0–5.0 mm). Accordingly, it would be possible to have large distance between glass slides to inject the solutions and to create thicker bolus. However, this may cause difficulty in achieving cross‐linked uniformity due to the thermal diffusion during cross‐linking reaction and affect the physical characterization of the bolus. Second, we did not evaluate the degree of adhesion to the soft areas (e.g., breast). Because it was difficult to adhere to soft areas owing to the amount of PCL polymer deformation, the 3D bolus may have better adhesion to the soft areas. To address these limitations, additional evaluation is required in the future.

## 5 CONCLUSION

We developed a thermoset shape memory bolus made from PCL polymer and evaluated the physical and dosimetric characterization. The developed bolus had almost perfect shape memory characteristics and conformally adhered to the body surface. The crystallization time was approximately 1.5 min, which was clinically acceptable. In addition, dosimetric characterization was equivalent to 3D boluses. Therefore, the shape memory bolus has excellent adhesion to the body surface, can be created in a short time, can be reused, and is useful as a bolus for radiotherapy.

## CONFLICT OF INTEREST

The authors have no relevant conflict of interest to disclose.
